# Influence of tourniquet use in primary total knee arthroplasty with drainage: a prospective randomised controlled trial

**DOI:** 10.1186/s13018-017-0683-z

**Published:** 2017-11-14

**Authors:** Kai Zhou, Tingxian Ling, Haoyang Wang, Zongke Zhou, Bin Shen, Jing Yang, Pengde Kang, Fuxing Pei

**Affiliations:** Department of Orthopaedics, West China Hospital of Sichuan University, Chengdu, 610041 China

**Keywords:** Prospective randomised controlled trial, Total knee arthroplasty, Tourniquet

## Abstract

**Background:**

We aimed to compare the effect of tourniquet use or lack of it on recovery following uncomplicated primary total knee arthroplasty (TKA).

**Methods:**

In a prospective randomised double-blinded study, 150 patients undergoing primary TKA were assigned to either a tourniquet or non-tourniquet group. At the early phase, 3 and 6 months after surgery, an independent observer assessed the primary outcome measure (i.e. total blood loss) and secondary outcome measures (i.e. wound complications, visual analogue scale pain score and knee range of motion).

**Results:**

The tourniquet group exhibited reduced intraoperative blood loss (215.7 ± 113.7 ml vs. 138.6 ± 93.9 ml, *P* < 0.001) and shorter operating time (77.2 ± 14.5 min vs. 82.0 ± 12.7 min, *P* = 0.038). However, the non-tourniquet group showed less postoperative blood loss (180.2 ± 117.0 ml vs. 253.7 ± 144.2 ml, *P* = 0.001) and drainage volume (89.2 ± 66.3 ml vs. 164.5 ± 97.8 ml, *P* = 0.004), less thigh pain (all *P* < 0.001) in the initial 3 weeks, better knee range of motion (ROM) in the initial 3 days (day 1 81.6 ± 17.1 vs. 75.95 ± 14.55, *P* = 0.036; day 3 99.8 ± 13.7 vs. 93.95 ± 11.15, *P* = 0.005) and fewer wound tension vesicles (10.3 vs. 29.2%, *P* = 0.005). Earlier straight-leg raising (4.6 ± 3.8 h vs. 6.4 ± 4.3 h, *P* = 0.01) and shorter length of stay (6.3 ± 1.7 days vs. 7.1 ± 1.9 days, *P* = 0.001) were found in the non-tourniquet group. Similar total blood loss and blood transfusion rate were observed for both groups. All other parameters revealed no significant differences.

**Conclusions:**

Our study suggests that a non-tourniquet TKA would lead to early rehabilitation without increasing side effects.

**Trial registration:**

Chinese Clinical Trials Registry, ChiCTR-IOR-16007851, 1/29/2016

## Background

Tourniquets are commonly used in total knee arthroplasty (TKA) to provide better visualisation and facilitate cementing techniques [1]. Although widely accepted, tourniquet-related complications have been reported in the literature, including wound complications, thigh pain, limb swelling, nerve palsy and muscle myofibril injury [2, 3]. In view of the tourniquet-related complications, some surgeons would like to perform TKA without a tourniquet [4, 5].

Several randomised controlled trials (RCTs) have compared the effects of tourniquet and non-tourniquet technique in TKA, but they drew different conclusions, and the sample sizes were small [5–8]. It is not clear whether it is necessary to use a tourniquet during TKA with the advances in surgical techniques and perioperative managements. Moreover, more high-quality research is still needed to identify the effect of the tourniquet in Chinese patients. Therefore, we conducted a randomised, controlled, prospective trial in which the patients receiving primary TKA were divided into two groups according to whether a tourniquet was used or not used. We hypothesised that the tourniquet would decrease intraoperative blood loss and total blood loss, whereas the non-tourniquet group would experience a decrease in limb swelling and promotion of faster recovery during the early postoperative rehabilitative period.

## Methods

The study was approved by the Ethics Committee and Institutional Review Board of West China Hospital, Sichuan University, and informed patient consent was also obtained. The trial was registered in the Chinese Clinical Trials Registry (ChiCTR-IOR-16007851) and conducted at a single centre. One hundred fifty patients who were suffering from end-stage osteoarthritis or rheumatoid arthritis and scheduled for a primary unilateral TKA were recruited. The exclusion criteria were patients with prior surgery involving the femur or tibia, prior lower extremity fracture, coagulopathy and uncontrolled hypertension. Patients were enrolled from January 2016 to May 2016 in our hospital. The patients were randomly divided into a tourniquet group and a non-tourniquet group according to a computerised random sequence generator. The sequence was concealed until the interventions were assigned by a sealed envelope method in the operating room. The observers collecting the data after the surgeries were uninvolved in the experimental operations and were unaware of the intervention assignments.

All the operations were performed by the same surgeon using a Sigma fixed or rotating plant posterior-stabilised total knee prosthesis (PFC, Johnson & Johnson/DePuy, Warsaw, IN, USA). We made an anterior midline skin incision with a medial parapatellar approach, using intramedullary guides for the femur and using extramedullary guides for the tibia. All patients received a general anaesthetic with controlled hypotension [9]. High-pressure pulsatile lavage was used to clean the bone surfaces and soft tissues. We then pressurised the cement into the cancellous bone to ensure better cement interdigitation. At the end of the operation, an intra-articular drain was placed and retained less than 24 h after surgery.

Each patient received the same perioperative treatment strategies: tranexamic acid (TXA), pain control and rehabilitation. TXA was given at the initiation of the surgery and just before closure [10]. Multimodal postoperative pain management and accelerated physical therapy were performed as previously described [11]. For thrombosis prophylaxis, oral rivaroxaban (10 mg per day) was initiated 12 h after the operation and was continued for 2 weeks. After recovery from anaesthesia, quadriceps femoris muscle isometric contraction was immediately started, and rehabilitation began on the first postoperative day, including muscle power training and passive and active range-of-motion (ROM) training.

The primary outcome was perioperative blood loss. The blood volume of each patient was calculated according to a formula that considers patient weight, height and sex [12]. In addition, intraoperative blood loss, postoperative blood loss, hidden blood loss, drainage volume and blood transfusion were also recorded. The increased weight of the gauzes plus the volume in the aspirator bottle excluding rinse represented the intraoperative blood loss. Hidden blood loss was calculated according to a formula published by Gross [13]. The postoperative blood loss was calculated by measuring the drainage volume and by weighing the dressings. The secondary outcome measures were evaluated during hospitalisation (ability to achieve a straight-leg raise; visual analogue scale [VAS] pain score; transfusion requirements; thigh, calf and knee swelling; ROM; hospital for special surgery [HSS] score; length of stay) and 3 weeks to 6 months following surgery (HSS scores, ROM, VAS pain score). Postoperative VAS scores were generated from thigh pain, knee rest and active pain. Thigh circumference (10 cm proximal to the superior border of the patella), knee circumference (midpoint of the patella) and calf circumference (20 cm proximal to the medial malleolus) were recorded to assess the degree of limb swelling. Knee active ROM was measured in a supine position by using a long-arm goniometer. The time to achieve a straight-leg raise after anaesthesia recovery was recorded. The decision to transfuse was based on the clinical symptoms of anaemia and laboratory results. Blood transfusion was given to patients whose postoperative haemoglobin (Hb) was less than 8 or 9 g/dl along with symptoms of anaemia such as tachycardia, dyspnoea, hypotension and light-headedness. Special attention was paid to whether symptomatic pulmonary embolism (PE) or deep vein thrombosis (DVT) occurred. DVT was detected by a regular bilateral lower extremity deep venous colour Doppler ultrasound examination. Other complications, such as erythema/ecchymosis, wound tension vesicle or superficial and deep infection were also recorded.

### Statistical analysis

Quantitative data are presented as the mean and standard deviation (SD); categorical variables are reported as proportions. Differences in continuous variables between groups were evaluated with Student’s *t* test or Mann-Whitney *U* test, depending on the distribution characteristics of the data. A chi-square test or Fisher exact test for difference in proportions was used to estimate the differences between groups in categorical variables. Statistical significance was set at *P* < 0.05. Statistical analysis was completed using SPSS 17.0.

## Results

### Participant flow and baseline data

**Fig. 1:**
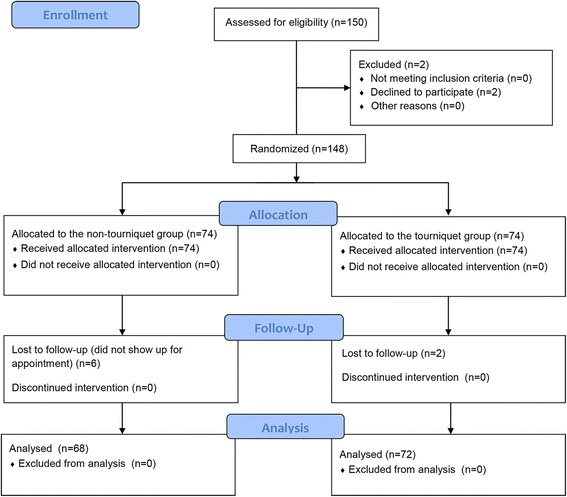
Patient flowchart

One hundred fifty participants were assessed for eligibility. Two were excluded for refusing to participate. After randomisation, 74 patients did not receive the limb tourniquet and comprised the non-tourniquet group (group A) and 74 patients received the limb tourniquet and constituted the tourniquet group (group B). Six patients in group A and two patients in group B were lost to follow-up, leaving 68 patients in the non-tourniquet group and 72 patients in the tourniquet group (Fig. 1). Baseline demographics, preoperative knee function, preoperative visual analogue scale (VAS) scores and clinical data are presented in Table 1. The two groups were equally matched by age, sex and body mass index (BMI). No significant differences were found between the two groups with regard to preoperative knee HSS scores, ROM, VAS scores and preoperative haemoglobin level.

**Table 1:** Preoperative demographics

Demographics	Non-tourniquet	Tourniquet	*P* value
Number of patients	68	72	–
Age (years)	69.1 ± 7.6	66.8 ± 8.6	0.095
Gender (female/male)	61/7	59/13	0.19
Diagnosis (OA/RA)	52/16	50/22	0.35
BMI (kg/m^2^)	25.7 ± 3.4	26.1 ± 4.1	0.53
Preoperative HSS score	47.7 ± 11.8	49.6 ± 12.3	0.351
Preoperative knee ROM	85.3 ± 27.5	89.5 ± 22.7	0.323
Preoperative haemoglobin (g/dl)	125.1 ± 12.9	123.8 ± 14.9	0.581
Preoperative VAS score			
Thigh pain	0.1 ± 0.6	0.0 ± 0.5	0.284
Knee rest pain	0.7 ± 1.3	0.5 ± 1.5	0.399
Knee active pain	5.5 ± 1.3	5.7 ± 1.6	0.417

Data are shown as mean ± standard deviation or numbers (%). Body mass index (BMI) equals weight in kilogrammes divided by the square of the height in metres

*OA* osteoarthritis, *RA* rheumatoid arthritis, *ROM* range of motion, *VAS* visual analogue scale, *HSS* hospital for special surgery

### Haemodynamic changes

The mean operating time in group B (77.2 ± 14.5 min) was significantly shorter than that in group A (82.0 ± 12.7 min) (*P* = 0.038, Table 2). Group A had an increased intraoperative blood loss (215.7 ± 113.7 ml vs. 138.6 ± 93.9 ml, *P* < 0.001), but postoperative blood loss (180.2 ± 117.0 ml vs. 253.7 ± 144.2 ml, *P* = 0.001) and drainage volume (89.2 ± 66.3 ml vs. 164.5 ± 97.8 ml, *P* = 0.004) were reduced when compared to group B. There was no significant difference in total blood loss between the groups (389.2 ± 178.3 ml vs. 374.5 ± 165.3 ml, *P* = 0.613). Blood transfusion was necessary for three patients in group A (4.4%), whereas eight patients in group B (11.1%) needed it, although the difference was not significant (*P* = 0.141, Table 2).

**Table 2:** Perioperative blood loss and allogeneic blood transfusion

	Non-tourniquet	Tourniquet	*P* value
Operating time (min)	82.0 ± 12.7	77.2 ± 14.5	0.038
Total blood loss (ml)	389.2 ± 178.3	374.5 ± 165.3	0.613
Intraoperative blood loss (ml)	215.7 ± 113.7	138.6 ± 93.9	< 0.001
Postoperative blood loss (ml)	180.2 ± 117.0	253.7 ± 144.2	0.001
Drainage volume (ml)	89.2 ± 66.3	164.5 ± 97.8	0.004
Blood transfusion (num./%)	3 (4.4%)	8 (11.1%)	0.141

Data are shown as mean ± standard deviation or numbers (%)

### Clinical outcomes and complications

**Fig. 2:**
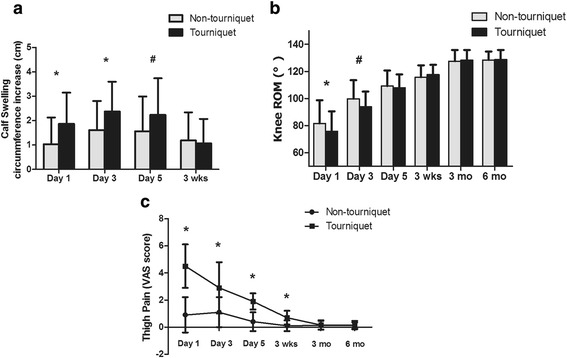
Difference between the two groups regarding postoperative calf swelling (**a**) and knee ROM (**b**) and knee active pain (**c**)

The differences in VAS score of thigh pain between the groups in the initial 3 weeks were statistically significant (all *P* < 0.001, Fig. 2a). More calf circumference increase was observed in group B at postoperative day 1 (1.87 ± 1.28 vs. 1.03 ± 1.1 cm, *P* < 0.001), day 3 (1.87 ± 1.28 vs. 1.03 ± 1.1 cm, *P* < 0.001) and day 5 (2.24 ± 1.50 vs. 1.5 ± 1.4 cm, *P* = 0.007, Fig. 2b). In the first 3 days after the operation, the patients in group A performed a better knee ROM (day 1 81.6 ± 17.1 vs. 75.95 ± 14.55, *P* = 0.036; day 3 99.8 ± 13.7 vs. 93.95 ± 11.15, *P* = 0.005, Fig. 2c); thereafter, the differences were no longer significant (all *P* > 0.05). A shorter length of stay after surgery (6.3 ± 1.7 days) and an earlier straight-leg raising (4.6 ± 3.8 h) were found in group A (*P* = 0.001, *P* = 0.01, Table 3). Analysis of postoperative knee function did not show any significant differences between the two groups with respect to the knee HSS score (discharge, 3 months, 6 months postoperative). The knee active pain VAS scores and thigh and knee circumference increase of the two groups at all observed time points were also not significantly different (all *P* > 0.05). Additional complications and side effects were noted for both groups and are shown in Table 3. Seven patients (10.3%) had wound tension vesicle in group A, and 21 (29.2%) in group B, and the difference was significant (*P* = 0.005). No significant differences were found in relation to wound erythema/ecchymosis, superficial wound infection or DVT. In addition, no deep prosthesis infection or PE was encountered in our study.

**Table 3:** Clinical outcomes and complication comparison

Parameters	Non-tourniquet	Tourniquet	P value
Length of stay	6.3 ± 1.7	7.1 ± 1.9	0.001
Straight-leg raising (num./%)	4.6 ± 3.8	6.4 ± 4.3	0.01
HSS score			
Discharge	66.6 ± 8.1	65.4 ± 7.4	0.359
3 months post-op	82.5 ± 4.5	81.6 ± 4.4	0.231
6 months post-op	89.8 ± 4.9	90.7 ± 4.5	0.257
Erythema/ecchymosis (num./%)	9 (13.2%)	15 (20.8%)	0.233
Tension vesicle (num./%)	7 (10.3%)	21(29.2%)	0.005
Superficial infection (num./%)	3 (4.3%)	5 (6.9%)	0.763
DVT (num./%)	0	2	0.497

Data are shown as mean ± standard deviation or numbers (%)

*DVT* deep vein thrombosis

## Discussion

The most important finding of the present study is that non-tourniquet TKA with wound drainage could decrease postoperative blood loss, thigh pain, calf swelling and wound tension vesicle. Furthermore, performing TKA without a tourniquet could promote early functional recovery with no additional side effects.

A tourniquet is mainly used to reduce intraoperative blood loss and to achieve better visualisation in TKA. It is reported that 58% of the members of the American Association of Hip and Knee Surgeons (AAHKS) use a tourniquet for TKA [14]. Although widely used, tourniquet-related complications have been reported in the literature, including soft-tissue and muscle damage, injury of calcified vessels, limb swelling, nerve injury and paralysis [15–17].

Notably, the tourniquet controls intraoperative blood loss but cannot stop postoperative blood loss or decrease overall blood loss [5]. Our study also demonstrated that the tourniquet use could decrease operation time and intraoperative blood loss, but there were no benefits in postoperative blood loss or total blood loss. The lost blood could escape into the soft tissue, potentially resulting in limb swelling, which would contribute to thigh pain, and additional swelling may hinder patients’ early postoperative function rehabilitation and increase the soft-tissue tension [18]. Similarly, our data suggested a higher level of thigh pain and calf swelling in the initial days after the operation in the tourniquet patients, and the patients without tourniquet presented better clinical outcomes. After the tourniquet was released, the ischemia-reperfusion (I-R) injury would occur when blood perfusion was re-established [19], and it would increase the risk of DVT due to stasis of venous blood in the lower limb and possible damage to blood vessels [17]. In our study, DVT or PE was not detected in either of the two groups. Chemical prophylaxis with rivaroxaban and early physiotherapy may be of great benefit. Two meta-analyses also found no difference in DVT or PE occurrence with the use of the tourniquet application during TKA surgery [16, 17].

Considering these above tourniquet-related complications, much effort has made to optimise tourniquet use by reducing the pressure, changing the cuff size and shortening the time of use. Olivecrona et al. [20] in a randomised study demonstrated that reduced tourniquet cuff pressure would lead to a lower rate of postoperative infections and wound complications. Ejaz et al. reported that the short-duration tourniquet was associated with better clinical outcomes, less pain and less limb swelling during the early stage of rehabilitation. Similar results were reported by Zhang et al. [21]. However, some knee surgeons, considering the side effects, would like to operate without a tourniquet. Especially for patients with popliteal artery calcification, no palpable pedal pulses and known peripheral vascular disease, it is safer to perform TKA without a tourniquet [22]. In addition, numerous studies reported no significant difference in the total blood loss with or without tourniquet [21, 23]. Zhang et al. [21] evaluated 13 RCTs including 698 knees in a meta-analysis. They found a mean 198 ml more intraoperative blood loss without a tourniquet. However, no difference was found in total blood loss. At the same time, postoperative knee ROM in the non-tourniquet group was 10.41° more than that in the tourniquet group at the early stage. These benefits may result from decreasing direct compression on tissue and reperfusion injury. Moreover, a better surgical haemostasis could be achieved because the bleeding structure is clearly observed without a tourniquet.

Theoretically, however, the absence of a tourniquet does obscure the bloodless surgical field and it requires more meticulous haemostasis during exposure and soft-tissue release because there may be blood, debris and fat on the bone surface, compromising cementation [5].

In our study, the comprehensive bleeding control method was used to minimise soft-tissue and bone surface bleeding to ensure the bone-cement interface fixation. First, our patients received a general anaesthetic with controlled hypotension, and mean arterial pressure was maintained between 60 and 70 mmHg. It helps greatly to reduce intraoperative bleeding without influencing blood supply to important organs [9]. Second, before opening the joint capsule, we injected 40 ml of 0.5% epinephrine along the incision to reduce soft-tissue bleeding. After finishing bone cutting in the non-tourniquet TKAs, hydrogen peroxide was used to remove coagulated blood and fat on the bone surfaces before using high-pressure pulsatile lavage. Our data suggest that the comprehensive bleeding control in non-tourniquet TKAs could lead to acceptable total blood loss.

One of the strengths of our study is that it is a randomised, controlled trial of a non-selected study population with few exclusion criteria. Another is the relatively large sample size we provided to prove that not using a tourniquet during TKA could yield fast recovery without increasing complications. The limitation of our study is the lack of long-term clinical and radiographic results. Future follow-up studies focusing on the clinical and radiographic outcomes might be meaningful.

## Conclusion

Based on our data, following the non-tourniquet TKA, postoperative blood loss, calf swelling, wound tension vesicle and thigh pain in the initial postoperative period would be reduced. Moreover, better knee ROM, shorter straight-leg raising time and shorter length of stay could also be attained. Our study suggests that a TKA performed without a tourniquet is safe and would lead to early rehabilitation.

## Abbreviations

BMI: Body mass index
DVT: Deep vein thrombosis
HSS: Hospital for special surgery
PE: Pulmonary embolism
ROM: Range of motion
TKA: Total knee arthroplasty
TXA: Tranexamic acid
VAS: Visual analogue scale
